# Whole-Brain *In-vivo* Measurements of the Axonal G-Ratio in a Group of 37 Healthy Volunteers

**DOI:** 10.3389/fnins.2015.00441

**Published:** 2015-11-27

**Authors:** Siawoosh Mohammadi, Daniel Carey, Fred Dick, Joern Diedrichsen, Martin I. Sereno, Marco Reisert, Martina F. Callaghan, Nikolaus Weiskopf

**Affiliations:** ^1^Department of Systems Neuroscience, University Medical Center Hamburg-EppendorfHamburg, Germany; ^2^Wellcome Trust Centre for Neuroimaging, UCL Institute of Neurology, University CollegeLondon, UK; ^3^Birkbeck/UCL Centre for NeuroImaging, Birkbeck CollegeLondon, UK; ^4^UCL Institute of Cognitive Neurology, University College LondonLondon, UK; ^5^Medical Physics, Department of Radiology, University Medical Center FreiburgFreiburg, Germany; ^6^Department of Neurophysics, Max Planck Institute for Human Cognitive and Brain SciencesLeipzig, Germany

**Keywords:** magnetization transfer imaging, g-ratio, *in-vivo* histology, myelin volume fraction, fiber volume fraction, diffusion MRI, DTI, multi-parameter mapping

## Abstract

The g-ratio, quantifying the ratio between the inner and outer diameters of a fiber, is an important microstructural characteristic of fiber pathways and is functionally related to conduction velocity. We introduce a novel method for estimating the MR g-ratio non-invasively across the whole brain using high-fidelity magnetization transfer (MT) imaging and single-shell diffusion MRI. These methods enabled us to map the MR g-ratio *in vivo* across the brain's prominent fiber pathways in a group of 37 healthy volunteers and to estimate the inter-subject variability. Effective correction of susceptibility-related distortion artifacts was essential before combining the MT and diffusion data, in order to reduce partial volume and edge artifacts. The MR g-ratio is in good qualitative agreement with histological findings despite the different resolution and spatial coverage of MRI and histology. The MR g-ratio holds promise as an important non-invasive biomarker due to its microstructural and functional relevance in neurodegeneration.

## Introduction

Understanding the normal and diseased human brain crucially depends on reliable knowledge of its anatomical microstructure. An important microstructural property is the g-ratio of fibers, which is defined as the ratio of the axonal diameter to the outer fiber diameter including the myelin sheath (Rushton, 1951; Hodgkin, 1964; Stikov et al., 2011). The g-ratio is related to the conduction velocity of nerve fibers (e.g., Rushton, 1951; Johansen-Berg and Behrens, 2009) and thus of significant functional relevance. For example, g-ratio maps could be used in conjunction with structural connectivity maps (Knösche and Tittgemeyer, 2011) to assess the importance of connective pathways. The g-ratio can change due to functional stimulation (Gibson et al., 2014) and thus might be an important indicator of structural plasticity (Zatorre et al., 2012). Clinical research and diagnosis would also benefit from measuring this key property of fiber pathways. For example, the cortical g-ratio in multiple sclerosis patients is higher as compared to cortical g-ratio in controls, probably because of de- and re-myelination processes (Albert et al., 2007).

Until recently, information about the g-ratio distributions in white matter has been accessible only by invasive methods such as *ex-vivo* electron microscopy (Hildebrand and Hahn, 1978). *In-vivo* MRI-based measurement of the g-ratio on a voxel-by-voxel level would be highly desirable. Stikov et al. proposed a non-invasive *in-vivo* MR-based “aggregate” g-ratio (Stikov et al., 2011) - in the following denoted “MR g-ratio.” Measurements of the g-ratio made with invasive methods such as electron microscopy allow the g-ratio of single axons to be measured. This is denoted the microscopic g-ratio. In contrast, the MR g-ratio framework measures the ensemble average of an underlying, unresolved, microstructural distribution of g-ratios - like many other voxel-wise quantitative MRI metrics (Weiskopf et al., 2015). Making a strong assumption about the g-ratio being constant within a voxel, Stikov et al. demonstrated via a geometrical plausibility argument (Stikov et al., 2011, 2015) that the MR g-ratio is related to the ratio of myelin and fiber volume fractions (MVF and FVF) within a given volume. To measure the MVF and FVF of the MR g-ratio, Stikov et al. (2011) initially used a quantitative magnetization transfer (MT) imaging method and the fractional anisotropy (FA) derived from diffusion tensor imaging (DTI). Although, the proposed MR g-ratio estimation method was demonstrated for the corpus callosum and showed great promise, several challenges for whole-brain high-resolution MR g-ratio mapping remained: (1) MR g-ratio estimates were limited to the corpus callosum, one of the few regions in the brain where the proposed relation between FVF and DTI-based FA maps was unique by avoiding crossing or fanning fibers, (2) susceptibility-related image distortions in the echo-planar-imaging (EPI) based DTI data were not corrected, which can lead to misalignment between MT and FA maps, (3) the acquisition used to determine the quantitative MT was rather time consuming, (4) until now the MR g-ratio was only investigated in a few volunteers (Melbourne et al., 2014; Stikov et al., 2015).

Two recent publications have further developed the MR g-ratio approach and made comparisons with *ex-vivo* measurements of the microscopic g-ratio. Using MRI and *ex-vivo* histology Stikov et al. (2015) compared the MR g-ratio in the corpus callosum of a cynomolgus macaque to the microscopic g-ratio. Furthermore, they measured the MR g-ratio over the whole brain for one healthy volunteer and one patient with multiple sclerosis using a beyond tensor model to estimate the FVF. Subsequently, West et al. (2015) showed, with *ex-vivo* histology measurements, that when a distribution of g-ratios are present within a voxel, the MR g-ratio is equal to the area-weighted root-mean-square of the microscopic g-ratios of individual fibers.

Despite these important recent advances in the MR g-ratio methodology, there is little known about the variation of the MR g-ratio within the population and across the brain's white matter.

In this study, we present an alternative, more time-efficient method that allows the spatial distribution of the MR g-ratio to be determined within the population and across the entirety of the brain's white matter. To improve our understanding of the MR g-ratio, we (a) implement a careful correction of susceptibility artifacts in diffusion MRI (dMRI) in order to avoid bias in MR g-ratio maps, (b) map variation of the MR g-ratio across the entire brain in a group of 37 healthy volunteers, and (c) compare the population maps to known variance for the g-ratio from *ex-vivo* histology literature values (Hildebrand and Hahn, 1978; Liewald et al., 2014; Stikov et al., 2014a).

## Theory

### The microscopic and MR g-ratio

**Figure 1:**
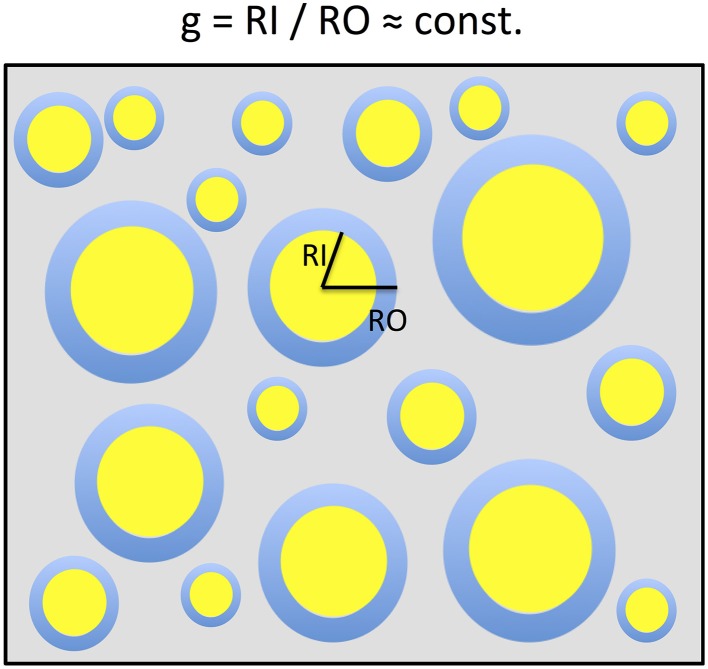
**A two-dimensional sketch of a voxel that includes myelinated axons that are arranged in parallel and have different axonal sizes but a constant g-ratio**. The g-ratio is the ratio of the inner (RI) and outer (RO) radii of a myelinated axon. For ensembles of myelinated axons with these properties, the MR g-ratio will be equal to the microscopic ensemble average of g-ratios. This sketch is based on the model of Stikov et al. (2011, 2014a,b).

In a simplified model a voxel in white matter can be subdivided into the volume occupied by myelinated axons and the extra-axonal volume fraction (EVF). When assuming that the myelinated axons can be described as parallel annular cylinders, the microscopic Fiber-Volume Fraction (FVF) and the Myelin-Volume Fraction (MVF) can be described in plane as nested two-dimensional circles (Figure 1). In this model, the mean FVF and MVF can then be calculated by summation over the axons within the white matter voxel.

(1) $$FVF=\frac{N}{A}\sum_{j=1}^{N_{\max}}\pi R_{O,j}^{2}P_{1}(R_{O,j})$$

and

(2) $$MVF=\frac{N}{A}\sum_{j=1}^{N_{\max}}\left(\pi R_{O,j}^{2}P_{1}(R_{O,j})-\pi R_{I,j}^{2}P_{2}(R_{I,j})\right)$$

with *R*_*O, j*_ and *R*_*I, j*_ being the outer and inner fiber radii, *P*_1_*(R*_*O, j*_*)* and *P*_2_*(R*_*I, j*_*)* the normalized probability of finding an axon with diameter *R*_*O, j*_ and *R*_*I, j*_in a voxel of area *A*, *N*_max_ the number of the last bin in the distributions *P*_1_ and *P*_2_, and *N* the total number of axons within a voxel.

On the other hand, we can estimate the expectation value for the microscopic g-ratio (*g*_*j*_ for axon *j*) in the voxel given the probability distribution *P* of microscopic g-ratios:

(3) $$g^{\text{micro}}=\sum_{j=1}^{N_{\max}}P{\left(g_{j}\right)}^{*}g_{j}.$$

If, as in previous studies (Stikov et al., 2011, 2014b) we assume that the g-ratio is constant within the voxel [i.e., *g*_*j*_ ≈ *const.* (≡ *g*^*MR*^) for all *j*], a simple relation between FVF and MVF can be derived and Equation (2) becomes:

(4) $$\begin{array}{l} MVF=\frac{N}{A}\sum_{j=1}^{N_{\max}}{\left(\pi R_{O,j}^{2}P_{1}(R_{O,j})-{\left(g^{\text{MR}}\right)}^{2}\pi R_{O,j}^{2}P_{2}(gR_{O,j})\right)}^{\left(i\right)}\\ \;=\frac{N}{A}(1-{\left(g^{\text{MR}}\right)}^{2})\sum_{j=1}^{N_{\max}}P_{1}(R_{O,j})\pi R_{O,j}^{2} \end{array}$$

where in (i) we replaced *P*_2_(*gR*_*O, j*_) by *P*_1_(*R*_*O, j*_), based on the argument that there is a one-to-one correspondence between each *R*_*I*_,_*j*_ and *R*_*O, j*_ given by *R*_*I*_,_*j*_*/R*_*O*_,_*j*_ = *g*^*MR*^ (see Figure 1).

Combining Equation (1) and (4) relates the MR g-ratio to the MVF and FVF:

(5) $$g^{\text{MR}}=\sqrt{1-MVF/FVF}.$$

Thus, in the case of a constant g-ratio in the voxel, the expectation value of the g-ratio (Equation 3) can be directly described by the MVF and the FVF (Equation 5). Note that the MR g-ratio can deviate from the microscopic g-ratio (this will be further addressed in the discussion section).

The MR g-ratio can also be related to a distribution of fibers with different g-ratios as recently shown by West et al. (2015). In this “revised g-ratio model,” the MR g-ratio equals the area-weighted root-mean-square of microscopic g-ratios of individual fibers.

## Methods

### Subjects

Thirty eight healthy volunteers (28 female, 10 male, age ± standard deviation: 23 ± 2.8 year) participated in the study approved by the local ethics committee, after giving written informed consent. One subject (male) was removed from the analysis because of poor dMRI data quality at the genu and the splenium of the corpus callosum (assessed by visual inspection of the tensor-fit error and orientation distribution function).

### Data acquisition

#### Diffusion

Experiments were performed on a 3T MAGNETOM Tim Trio MRI scanner (Siemens Healthcare, Erlangen, Germany) operated with a standard 32-channel radio-frequency (RF) head coil for receive and an RF body coil for transmission. Standard single-shell dMRI (Nagy and Weiskopf, 2008) data were acquired using the following parameters: 60 diffusion-weighted (DW) images (*b* = 1000 s/mm^2^), 6 T2-weighted images with low diffusion weighting (*b* = 100 s/mm^2^ images), 60 slices, 2.3 mm slice thickness, with no gap, 6/8 partial Fourier imaging in phase-encoding direction, 220 × 220 mm^2^ field-of-view (FoV), in-plane resolution 2.3 × 2.3 mm^2^, echo time of *TE* = 90 ms, acquisition time per slice = 170 ms (volume repetition time *TR* = 10.2 s), total acquisition time approximately 11 min. To correct for susceptibility-related image distortions, an additional low-diffusion weighted image with reversed phase-encoding direction and otherwise identical acquisition parameters were acquired (~1 min acquisition time). This resulted in a total acquisition time of about 12 min for each subject. Note that a slightly longer acquisition time per slice was chosen to ensure minimal table-vibration artifacts (for details see, Mohammadi et al., 2012a). Furthermore, we measured gradient-non-linearities during the diffusion weighting using a brain-sized water phantom (for details see Mohammadi et al., 2012b). For our scanner the gradient-non-linearities were less than 2% within the water phantom and thus negligible as a source for bias (see, Bammer et al., 2003; Mohammadi et al., 2012b).

#### Magnetization transfer imaging

For each subject a whole-brain quantitative multi-parameter mapping (MPM) protocol (Dick et al., 2012; Weiskopf et al., 2013) was acquired to estimate 0.8 mm isotropic magnetization transfer saturation (MT) maps. The protocol consisted of proton-density-weighted (PD), T1-weighted and MT-weighted fast-low-angle-single-shot (FLASH) acquisitions using the following parameters (adapted from Weiskopf et al., 2013): voxel size: 0.8 × 0.8 × 0.8 mm^3^, FoV 256 × 224 × 166 mm^3^, matrix 320 × 224 × 208, *TR* = 25.25 ms for the PD- and T1-weighted acquisitions and *TR* = 29.25 ms for the MT-weighted acquisition, excitation flip angle: 5° (PDw), 29° (T1w), or 9° (MTw). The MT weighting was achieved through application of a Gaussian RF pulse (4 ms duration, 220° nominal flip angle) applied 2 kHz off-resonance prior to non-selective excitation. The acquisition was accelerated by GRAPPA (with a parallel imaging factor of 2) in the phase-encoding direction as well as by a partial Fourier acquisition in the partition direction (with factor 6/8). To improve image quality, i.e., maximize signal-to-noise ratio (SNR) and minimize geometric distortion at the same time, eight gradient echoes were acquired with high readout bandwidth (460 Hz/pixel) after each excitation pulse. The total scanning time of the MPM protocol was approximately 37 min. Quantitative parameter maps were derived from the MPM protocol using bespoke MATLAB tools (The Mathworks Inc., Natick, MA, USA) implemented in a toolbox for voxel-based quantification (VBQ; Draganski et al., 2011; Weiskopf et al., 2013). The first six echoes for each of the three acquired weightings were averaged to increase the SNR. The resulting PDw, T1w, and MTw volumes were used to calculate maps of MT and R1 as described previously (Weiskopf et al., 2013). The MT map is a semi-quantitative measure of the magnetization saturation caused by the MT pre-pulse and the dynamics of the transfer between bound and mobile proton pools. Consequently, if direct saturation is kept low as in our implementation of the MT sequence, this magnetization transfer measure provides information about the macromolecular content of the microstructural environment and hence is a semi-quantitative measure for the bound-pool fraction (Helms et al., 2008). This differs from the commonly used MT ratio (MTR; percent reduction in steady state signal) by explicitly accounting for spatially varying T1 relaxation times and flip angles (Helms et al., 2008) and results in higher contrast in the brain than MTR (Helms et al., 2010). Additional minor corrections for flip angle inhomogeneity in the MT maps were applied as described in Weiskopf et al. (2013).

### Pre-processing of dMRI data

The dMRI data were preprocessed using the ACID toolbox. They were corrected for motion and eddy current artifacts (Mohammadi et al., 2010), and for susceptibility-related distortion artifacts using the HYperelastic Susceptibility artifact Correction method (Ruthotto et al., 2012, 2013). The dMRI data were de-noised using the position-orientation-adaptive-smoothing (POAS) method (Tabelow et al., 2015). The diffusion-tensor and its indices, i.e., Fractional Anisotropy (FA), Mean (MD), Axial (AD), and Radial Diffusivity (RD), were estimated using the ACID toolbox (Mohammadi et al., 2013a,b).

The Tensor Fiber Density (TFD) was calculated using the Freiburg Fibertools (Reisert et al., 2011, 2013). The principle of TFD relies on the assumption that fiber orientation distributions correctly provide relative fiber volume fractions (Raffelt et al., 2012). This assumption together with a fiber conservation law, which is inspired by fluid dynamics, is used to derive absolute fiber numbers up to one global factor (Reisert et al., 2013). The algorithm itself is based on solving a discretized partial differential equation.

Finally, to correct for any motion between the dMRI and MPM acquisitions, the low-b image of the susceptibility-corrected dMRI dataset was registered to the 3rd PD-weighted echo (*TE* = 7.11 ms) using a modality-independent rigid-body registration in SPM12. The transformation was applied to all DTI indices (i.e., TFD, FA, MD, AD, and RD).

### G-ratio estimation

To estimate the MR g-ratio as derived in the theory section, three innovations that ensured a faster and more robust acquisition of the MR g-ratio maps compared to recent approaches (Stikov et al., 2011, 2014a,b; Campbell et al., 2014) were introduced: (a) *MVF* was estimated from MT maps, which use multi-echo FLASH with high SNR efficiency and image quality (Weiskopf et al., 2013, 2014) and only requires a single MT-weighted acquisition, (b) *FVF* was calculated using the TFD (Reisert et al., 2013), which unlike other higher-order diffusion models can be directly estimated from a comparatively small single-shell dMRI dataset, (c) we corrected for susceptibility-related distortions in the dMRI data to improve alignment between dMRI and MT data. Based on these innovations, the MR g-ratio became:

(6) $$g=\sqrt{1-\text{MVF/FVF}}=\sqrt{1-\alpha^{\text{MT}}/\text{TFD}},$$

where the same normalization α = 0.1 was used for all subjects. This normalization factor was determined by normalizing the MR g-ratio to a literature value of *g* = 0.7 for the splenium for a single subject (m, age = 26) from the studied cohort. The value of *g*=0.7 has been observed for large-diameter axons (which appear more frequently in the splenium) via *ex-vivo* electron-microscope measurements in humans (Graf von Keyserlingk and Schramm, 1984). Note that the rescaling constant α accounts simultaneously for both the previously mentioned global correlation factor between TFD and FVF and deviations between MT and MVF.

### The effect of susceptibility-related image distortions on the estimated MR g-ratio map

To demonstrate the effect of susceptibility-related image distortions in dMRI on the MR g-ratio estimation, we calculated the MR g-ratio of a representative subject before and after correcting the dMRI data for susceptibility distortions.

### Spatial normalization for group MR g-ratio maps

To capture the inter-individual variation, the individual MR g-ratio maps were transformed into a common group space. To this end, DARTEL as implemented in SPM12 (Friston et al., 2006; Ashburner, 2007) was used to estimate the deformation fields. Then, these fields were applied to the white-matter segments to morph them into MNI space. Instead of using a standard VBM-style of approach, we used the VBQ method as implemented in SPM8 to minimize partial volume effects associated with smoothing of the different tissue compartments (Draganski et al., 2011). Once in MNI space, summary statistics for the cohort (mean: < *g*>, standard deviation: *stdg*_*subj*_ and coefficient of variation: *stdg*_*subj*_/ < *g*>) were calculated on a voxel-wise basis. Finally, a histogram of the mean g-ratio values across WM voxels was calculated.

### MR g-ratio in specific fiber tracts

The group-mean MR g-ratio and its inter-individual standard deviation were calculated in 13 probabilistic fiber tracts defined in the SPM anatomy toolbox (Eickhoff et al., 2005), out of which 6 fiber tracts are presented in this paper, namely: corticospinal tracts (ct), optic radiation (or), inferior occipitofrontal fasciculus (iof), superior longitudinal fasciculus (slf), cingulum (cing), fornix (forn)—for visualization see **Figure 6**. The fiber tracts were delineated in myelin-stained histological sections of ten human post-mortem brains (Bürgel et al., 1999, 2006) and spatially normalized to the brain of Colin Holmes in MNI space (Evans et al., 1993; Collins et al., 1994; Holmes et al., 1998). The probabilistic maps contained within the Anatomy toolbox show the probability of finding a particular tract in a voxel across the ten brains. To exclude voxels that were affected by partial volume effects or included a wide variety of tracts across subjects, tract specific ROIs were created based on the conjunction of the probabilistic map for each tract thresholded at 50% and the CoV-map thresholded at CoV < 0.3. Note that we present only 6 out of 13 tracts since the remaining 7 tracts contain less than one hundred voxels.

These probabilistic fiber tract atlases are provided in MNI space (Eickhoff et al., 2005), normalized to the brain of Colin Holmes (Evans et al., 1993; Collins et al., 1994; Holmes et al., 1998), and thus might show small spatial misalignments with respect to the DARTEL-template MNI space of our group of subjects. The following procedure was followed to spatially register the probabilistic fiber tract atlases to the DARTEL-template MNI space. First, the DARTEL template was transformed into MNI space, then, the Colin Holmes brain was normalized to the DARTEL template using spm_normalize as implemented in SPM12 (Ashburner and Friston, 2005). The resulting deformation field was applied to the probabilistic fiber tract atlases.

Finally, to compare the MR g-ratio within the brain to previous reported *ex-vivo* g-ratio values (Hildebrand and Hahn, 1978; Liewald et al., 2014; Stikov et al., 2014a), we used an ANOVA that tested whether the g-ratio in one tract was significantly larger than in any other tract, and with *post-hoc t*-tests we determined those tracts that were significantly smaller than the cortico-spinal tract (at a statistical threshold of *p* < 0.05).

In addition, the group-averaged MR g-ratio in 8 manually defined ROIs within the corpus callosum (**Figure 4B**) was calculated. To define the manual ROIs, the corpus callosum was split into 8 equidistant intervals along the anterior-posterior direction (see **Figure 4B**) to match an *ex-vivo* histology study of g-ratios in a macaque monkey corpus callosum (Stikov et al., 2014a). To minimize partial volume effects, the ROI masks were additionally constrained by excluding voxels for which the CoV was greater than 0.3. The remaining number of voxels in each thresholded corpus callosum ROI was larger than 35. The group-averaged MR g-ratio and its standard deviation in the corpus callosum ROIs were compared to the corresponding measures in the macaque monkey data. Note that to match the standard-deviation estimation for the ROI analysis to the *ex-vivo* macaque monkey data, we first calculated the standard deviation across the ROI for each individual and afterwards performed the group average.

The same tract and ROI analyses as described above were also performed for the MT, TFD, and FA maps. Finally, to assess the relation between TFD and MT we respectively averaged the TFD and MT values within each ROI/fiber tract. Then, we calculated the correlation coefficients between the averaged TFD and MT values across the group for each ROI/fiber tract and tested the significance of the correlation.

## Results

### The effect of susceptibility-related image distortions on the estimated MR g-ratio map

**Figure 2:**
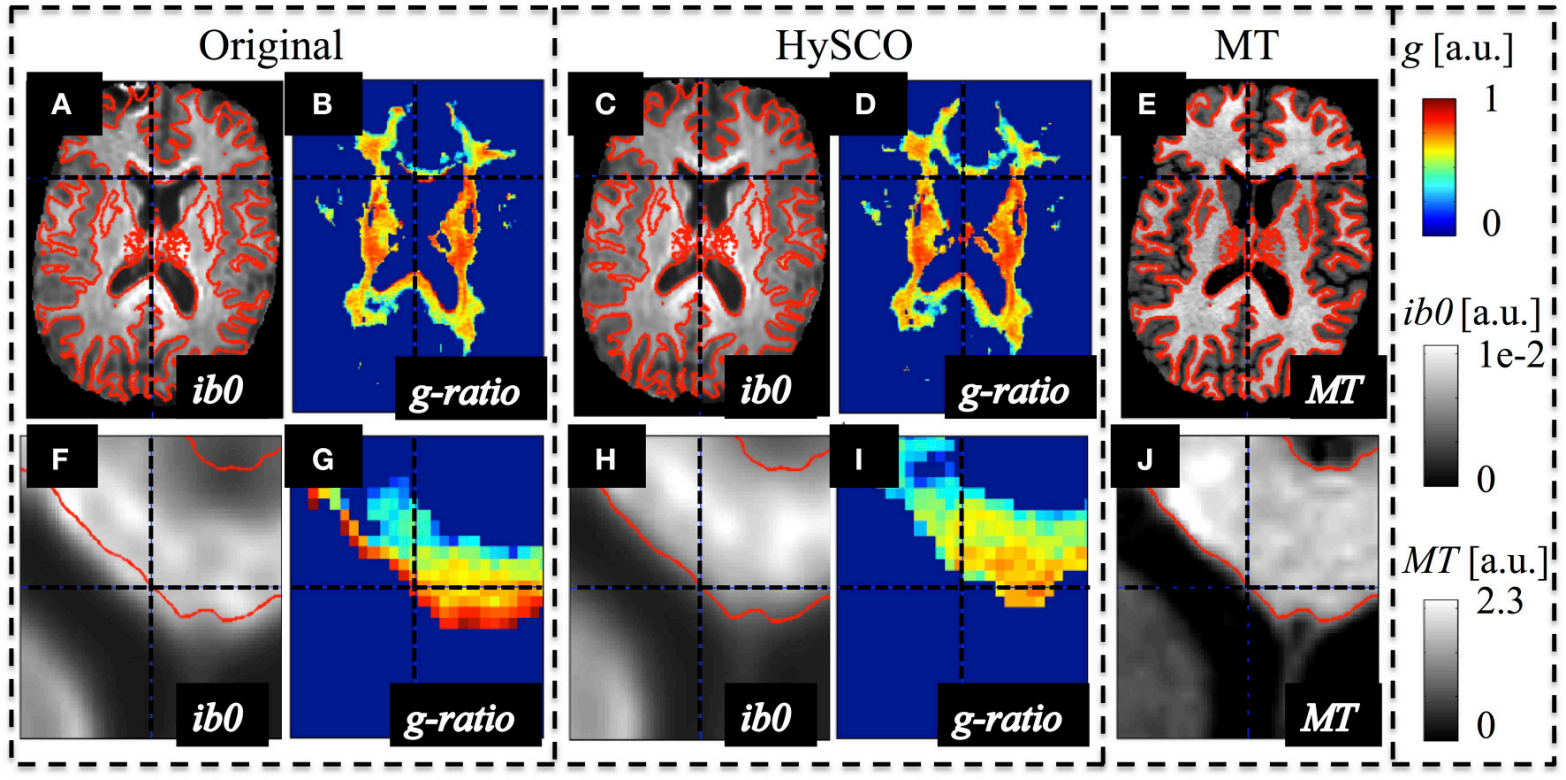
**Example of susceptibility-induced geometric distortions in the single-shell dMRI data and their effects on the estimated MR-based g-ratio map**. The MR g-ratio and contrast-inverted *b* = 0 maps (*ib0*) from the original **(A,B,F,G)** and susceptibility-distortion corrected dMRI data **(C,D,H,I)** of a representative subject were compared to the subjects' MT map **(E,J)**, which did not suffer from susceptibility artifacts. The spatial mismatch between anatomical structures in the single-shell dMRI and MT data (see contours in red) was strongly reduced after susceptibility correction. The susceptibility-related mismatches between uncorrected dMRI and MT maps led to a severe locally varying bias in the g-ratio maps [e.g., crosshair highlights one of the voxels with an unrealistic g ≈ 1 at the edge of the genu **(G)**].

The susceptibility-related distortions in the original dMRI data led to a spatial mismatch with respect to the MT data (see Figure 2). These distortions resulted in a localized bias in the calculated MR g-ratio maps (e.g., red edge with implausible g ≈ 1 at the genu, Figure 2G). Correction of these distortions via HySCO improved the spatial correspondence between the dMRI and MT data and removed the bias from the MR g-ratio maps, (Figure 2I).

### MR g-ratio in the population

**Figure 3:**
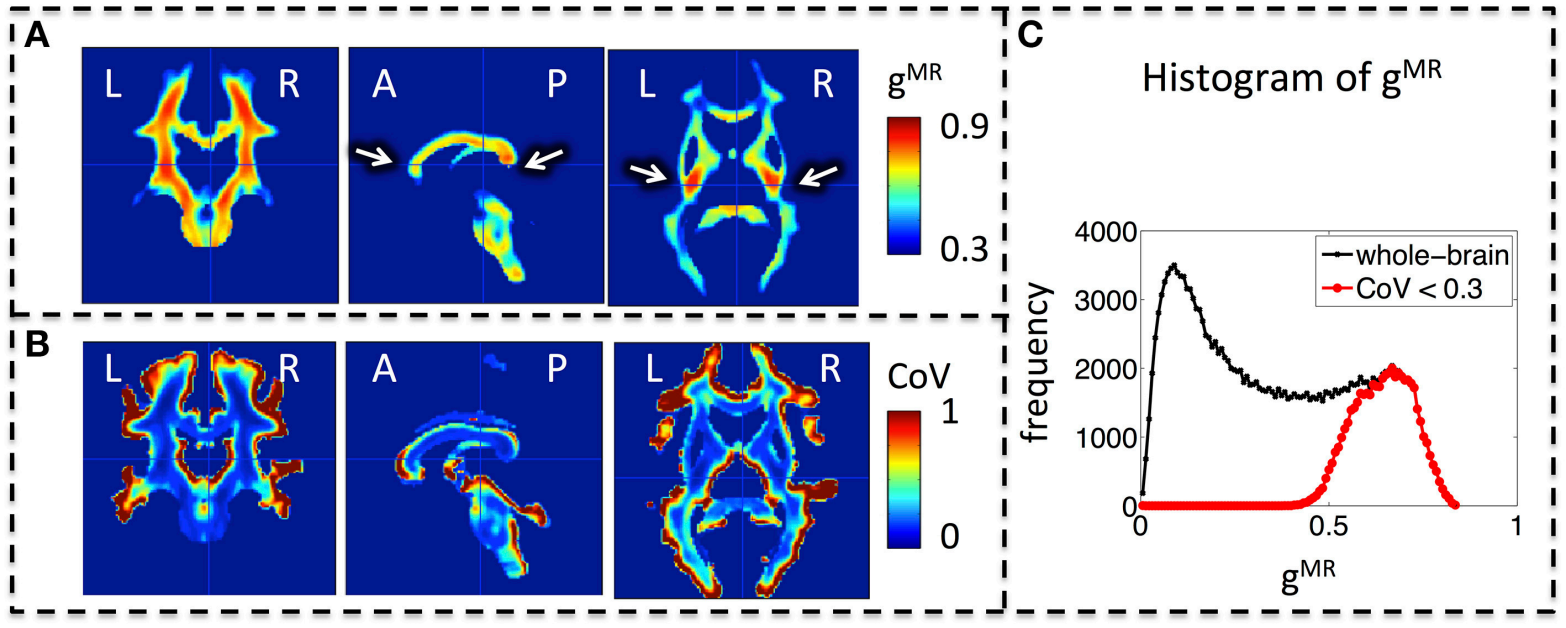
**Group MR g-ratio (g^MR^) maps**. **(A)** Map of mean MR g-ratio. The highest values occurred in the genu, midbody, and splenium of the corpus callosum and within the cortico-spinal tracts (arrows). **(B)** Map of coefficient of variation (CoV) of MR g-ratio. Inter-individual variation was particularly high at the edges of white matter pathways. **(C)** Histogram of mean MR g-ratio in white matter showed a broad distribution (0–0.8) when the whole brain was considered but became narrower (0.5–0.8) when the analysis was restricted to voxels with COV < 0.3. Abbreviations: L, left; R, right; A, anterior; P, posterior.

Whole-brain maps of the mean MR g-ratio across the group showed the highest g-ratio in the splenium of the corpus callosum and along the cortico-spinal tracts (Figure 3A, arrows). The largest inter-individual variation as measured by the CoV of the MR g-ratio occurred toward the edges of white-matter tracts (Figure 3B). The group-averaged MR g-ratio was mostly between 0.2 and 0.8, when the histogram was calculated over the whole white matter. When a histogram was calculated only using voxels for which the CoV was less than 0.3 the group-averaged MR g-ratio fell between 0.5 and 0.8 (Figure 3C).

**Figure 4:**
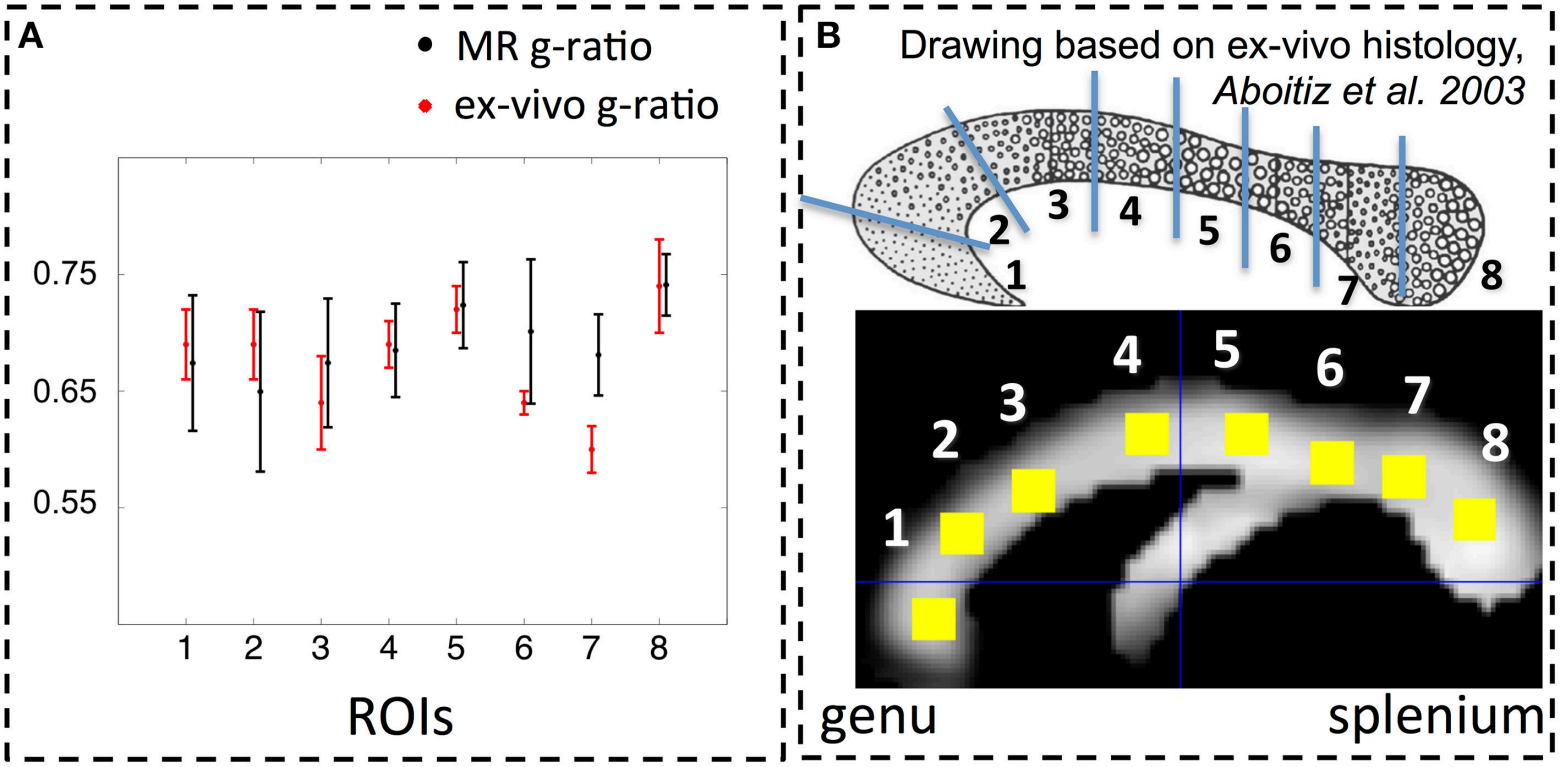
**(A)** Comparison between MR (black) and *ex-vivo* (red) g-ratio measures in the corpus callosum. **(B)** Following the parcellation of the corpus callosum by Stikov et al. (2014a) for *ex-vivo* histology (top row), we calculated the group mean MR g-ratio in 8 different region of interests (ROI, bottom row; error bars indicate standard deviation across ROI) and compared them to the corresponding *ex-vivo* g-ratio reported by Stikov et al. (2014b) (error bars indicate standard deviation across 3 different samples within each ROI). The MR g-ratio measures followed the trend of the *ex-vivo* measurements **(A)**. Note that the *ex-vivo* measurements of the g-ratio were obtained from a macaque monkey.

**Figure 5:**
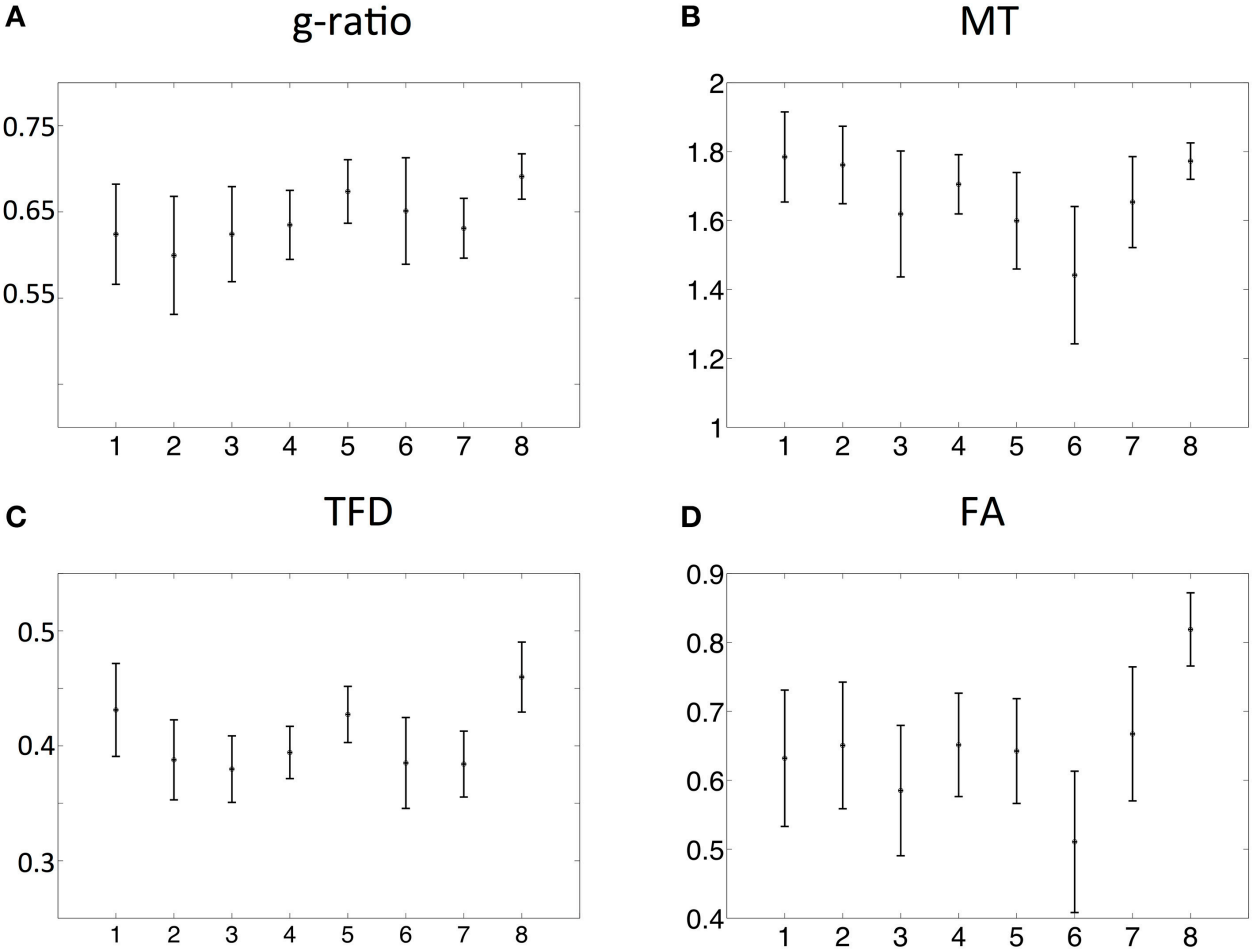
**Comparison of MR g-ratio (A) to the MT (B), TFD (C), and FA (D) values: the group mean and the standard deviation within the corpus callosum region-of-interests (ROIs, defined in Figure 3) were compared**. The TFD and FA values followed the trend of the MR g-ratio values in the corpus callosum.

The spatial dependence of the MR g-ratio in the corpus callosum was generally in line with the observations from *ex-vivo* electron microscopy measurements in macaque tissue samples (Stikov et al., 2014a). In particular, peak values were observed in the genu, midbody, and splenium of the corpus callosum for both measurements (Figure 4A). However, there were also slight deviations between *ex-vivo* and MR g-ratio measurements, e.g., their minimum was slightly shifted (ROI6 in our human data vs. ROI7 in the macaque data). Also the mean MR g-ratio in the corpus callosum was slightly lower in the human data, which may be explained by the choice of the normalizing constant α in the MR g-ratio calculation (Equation 6). To a certain degree, the trend in the *ex-vivo* g-ratio (Figure 5A) was reflected in the TFD (Figure 5C) and FA maps (Figure 5D) across the corpus callosum but not in the MT maps (Figure 5B).

**Figure 6:**
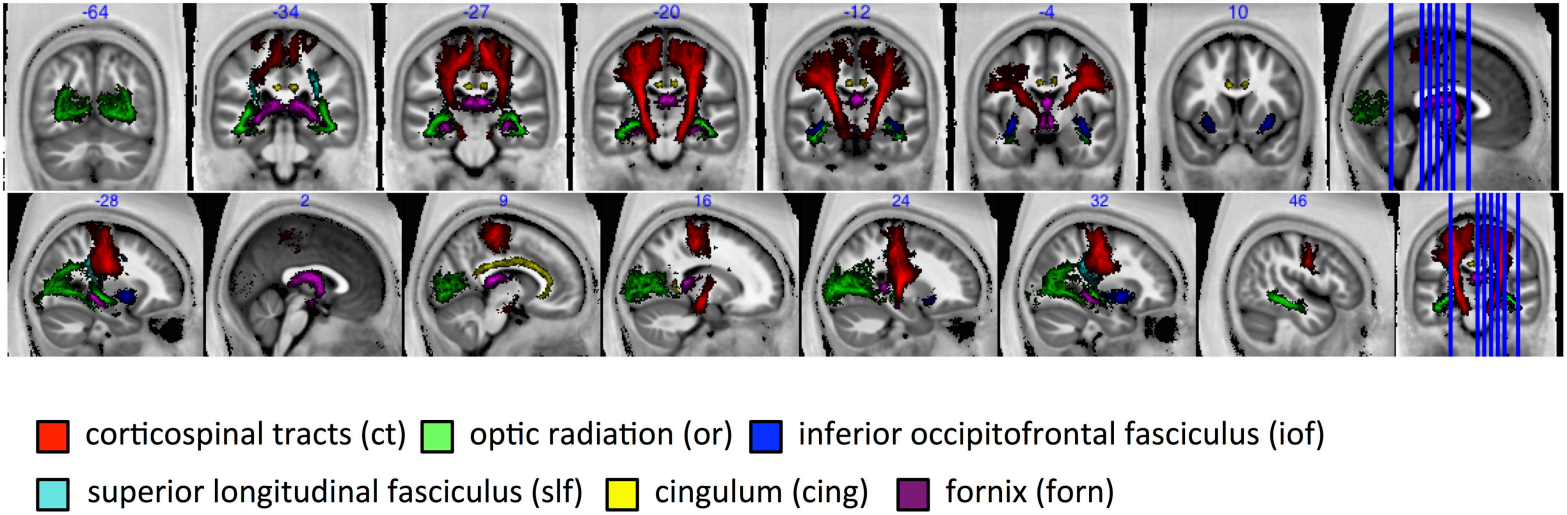
**For tract-specific analyses six major white-matter pathways were selected from the SPM anatomy toolbox (Eickhoff et al., 2005)**. ct, corticospinal tracts; or, optic radiation; iof, inferior occipitofrontal fasciculus; slf, superior longitudinal fasciculus; cing, cingulum; and forn, fornix.

**Figure 7:**
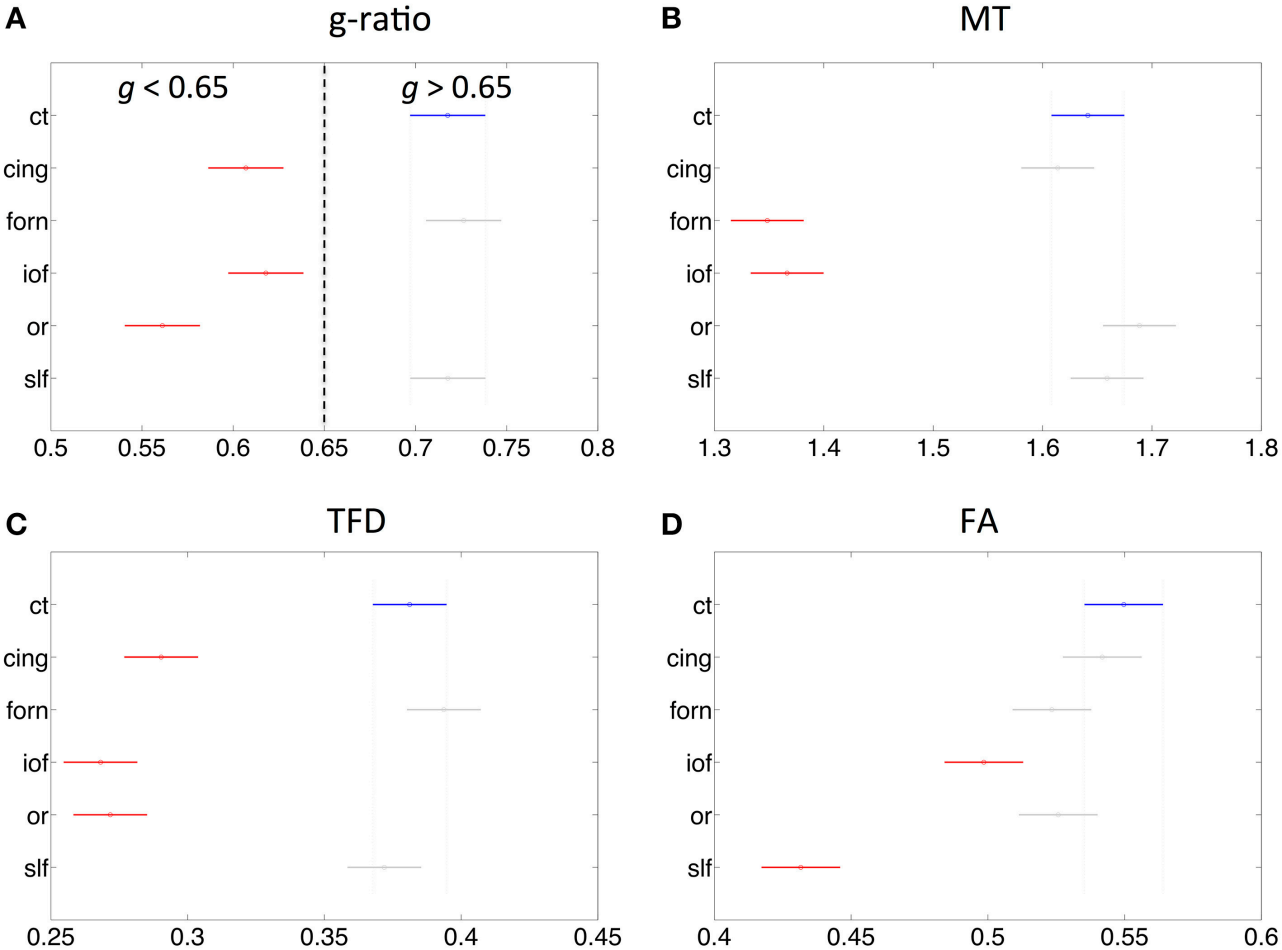
**Comparison of MR g-ratio to the (A) MT (B), TFD (C), and FA (D) values within six fiber tracts (as defined in Figure 6)**. Tracts in red show significant difference to cortico-spinal tract (blue). There is no obvious simple correspondence between MR g-ratio and the other quantitative MR values in these tracts, indicating that the MR g-ratio provides additional information over and above the other measures.

The fiber tract specific analysis of the MR g-ratio (based on ROIs defined in Figure 6) revealed that tracts can be divided into two categories (Figure 7A): tracts with g-ratios g > 0.65 (cortico-spinal tracts, fornix, and superior-longitudinal fasciculus) and tracts with g-ratios *g* < 0.65 (cingulum, inferior occipitofrontal fasciculus, and optic radiation). As compared to the cortico-spinal tracts, the g-ratio of the optic radiation, inferior-occipitofrontal fasciculus, and cingulum was significantly smaller (Figure 7A). The trend of the MR g-ratios across the presented tracts differed from that of the FA, TFD, and MT values (Figures 7B–D).

## Discussion

We have presented a novel method for calculating the MR g-ratio across the whole brain, which combines MT and standard single-shell dMRI data. This approach enabled us to investigate the variation of the MR g-ratio across prominent fiber pathways within the brain for a group of healthy subjects. The careful correction of susceptibility-related distortion artifacts in the dMRI data proved crucial for avoiding bias in the MR g-ratio estimates. The MR g-ratio showed a very similar spatial pattern along the rostral-caudal axis of the corpus callosum as *ex-vivo* electron microscopy measures in the macaque monkey (Stikov et al., 2014a). The variance in the measured MR g-ratio across major fiber tracts also agreed qualitatively with *ex-vivo* measurements in the literature (Graf von Keyserlingk and Schramm, 1984; Liewald et al., 2014).

### General limitations

Since this is the first whole-brain population study to measure the MR g-ratio, the possibilities for cross-validation are limited. A careful discussion of the general limitations of MR and *ex-vivo* based g-ratio measures and data is thus warranted.

Estimation of the MR g-ratio relies on the assumption that the g-ratio is approximately constant within a voxel. There are a number of situations where this assumption may be violated. It is known from *ex-vivo* literature that the g-ratio varies within white matter (e.g., Graf von Keyserlingk and Schramm, 1984; Guy et al., 1989; Tomasi et al., 2012). For example, the percentage of unmeylinated axons, i.e., those with a microscopic g-ratio of *g*^micro^ = 1, varies by more than 30% within the corpus callosum of the rhesus monkey (Lamantia and Rakic, 1990). As a consequence, g-ratio variation within a voxel (e.g., a multi-modal distribution of g-ratios with a myelinated and an unmyelinated pool) will lead to deviations between the MR g-ratio and the microscopic ensemble-averaged g-ratio. The recently published “revised g-ratio model” (West et al., 2015) addressed this limitation by showing that the MR g-ratio of a distribution of fibers corresponds to the fiber-area-weighted root mean square of the distribution.

Currently there is no widely accepted standard for state-of-the-art measurement of MVF or FVF. In fact, current methods that estimate the FVF (e.g., neurite orientation dispersion and density imaging or NODDI, Zhang et al., 2012, apparent fiber density or AFD, Raffelt et al., 2012, and tract-fiber density or TFD, Reisert et al., 2013) are likely to be biased due to limitations associated with the respective method. The NODDI estimates might be biased by the single-fiber approximation or the not validated assumptions about fixed diffusivity metrics (Jelescu et al., 2015). The apparent fiber density is weighted by the transverse relaxation time and thus difficult to interpret. The TFD metric is based on data with relatively low diffusion weighting (*b* = 1000 s/mm^2^) and thus can be affected by contributions from the extracellular water (e.g., simulations in Raffelt et al., 2011), The MVF, which is estimated from standard MT-saturation-based acquisitions such as ours or the quantitative MT approach of Stikov et al., can be biased because the MT-related exchange processes are not restricted to myelin macromolecules but also involve other macromolecules, e.g., those present in cell membranes. Alternative MRI methods that have been suggested to estimate the myelin content (e.g., using a multi-compartment T2-relaxation model, Mackay et al., 1994), have their own inter-linked limitations such as low SNR, long measurement time, low resolution and limited brain coverage.

Robust cross-validation of the MR g-ratio mapping with a gold standard is complicated, since rather little information and few *ex-vivo* studies on the microscopic g-ratio are published (e.g., Hildebrand and Hahn, 1978; Gibson et al., 2014; Stikov et al., 2014a). *Ex-vivo* studies suffer from their own specific limitations, e.g., shrinkage of the tissue that leads typically to an underestimation of axonal diameters (Assaf et al., 2013). Furthermore, the limited spatial coverage of *ex-vivo* techniques would hamper any whole-brain validation, even if the fixation issues and limited subject numbers were addressed.

### Comparison with *ex-vivo* histology

In order to allow for a comparison of the MR g-ratio outside the corpus callosum with histological studies, we refer to the positive relation between axonal diameter and g-ratio that saturates at larger fiber diameters (~1–4 μm) as frequently reported, e.g., in Hildebrand and Hahn (1978), Graf von Keyserlingk and Schramm (1984), Guy et al. (1989), Johansen-Berg and Behrens (2009), and Gibson et al. (2014). This allows for a comparison of our maps with more readily available histological measures of axonal diameter.

We found relatively large g-ratios (i.e., g ~ 0.7) in the cortico-spinal tracts, which is in accordance with the findings of Graf von Keyserlingk and Schramm (1984), who reported a g-ratio of >0.6 for axons with a diameter of >5 μm in the pyramidal tracts. We found that the MR g-ratio in the optic radiation, cingulum, and inferior-occipitofrontal fasciculus was significantly smaller (g ~ 0.6) than in the cortico-spinal tracts. In these fibers (g ~ 0.6), the g ratio is close to optimal, i.e., the value for which conduction velocity is maximal (Rushton, 1951). Finally, our observation that the MR g-ratio in the inferior occipitofrontal fasciculus is lower than in the superior longitudinal fasciculus is in line with the findings of Liewald et al. (2014), who demonstrated in 3 human brain tissue samples that the mean of the axonal diameter in the inferior-occipitofrontal fasciculus is lower than in the corpus callosum and/or in the superior-longitudinal fasciculus, i.e., the g-ratio is also expected to be lower according to the known positive relation between diameter and g-ratio (Graf von Keyserlingk and Schramm, 1984).

There is limited *ex-vivo* histology data available in the literature with which to compare our MR g-ratio measurements. In humans, the *ex-vivo* g-ratio has only been reported for the corticospinal tract (Graf von Keyserlingk and Schramm, 1984). A greater array of histological data is available from different species, e.g., the macaque monkey (Stikov et al., 2014a, 2015) and the guinea pig (Guy et al., 1989). Differences in anatomical microarchitecture between macaque monkey and human might explain why, despite observing the same general trend of high-low-high-low-high g-ratios along the rostral-caudal axis of the corpus callosum (Figure 4A), the location of the minima slightly differed between our MR g-ratio in humans and the *ex-vivo* g-ratio in the macaque.

### Clinically feasible MRI measures of MVF and FVF

There are various approaches to estimating the FVF (e.g., Jespersen et al., 2010; Reisert et al., 2013; Stikov et al., 2014a) and MVF (e.g., Müller et al., 2013; Stikov et al., 2015). So far, it is unclear which one is optimal.

Initially, Stikov et al. (2011) calculated the MR g-ratio within the corpus callosum using quantitative MT to estimate the MVF and FA to estimate the FVF. The MR g-ratio was restricted to regions of interest within the corpus callosum only, i.e., no whole brain g-ratio maps were presented. This was because the polynomial function relating FA and FVF, which was proposed by Stikov et al. (2011), was not expected to hold outside the corpus callosum due to an increased number of crossing fibers and other complex fiber configurations. Our results confirm this expectation, since we found a high correspondence between TFD and FA across the different regions of the corpus callosum but less so for the other prominent white matter tracts. Although, robust to more complex fiber geometries, one limitation of the TFD method is that it is a nonlocal metric (Reisert et al., 2013) and thus particularly sensitive to partial volume effects, i.e., it becomes unreliable toward the edge of the white matter. This likely explains the high CoV of the MR g-ratio maps (Figure 3B) toward the edge of white matter. To account for this limitation, we excluded all voxels from our analyses that had a CoV larger than 0.3. After removing the potentially unreliable estimates, the distribution of MR g-ratios within the white matter showed high correspondence to previously reported *ex-vivo* g-ratio distributions (compare Figure 3C and Guy et al., 1989).

Recently, an alternative method to estimate the MR g-ratio for the whole brain was proposed by Stikov et al. (2015). Their proof-of-concept study presented MR g-ratio maps for two human subjects (one patient and one control). Globally, we obtain similar g-ratio values (peak at 0.6) as Stikov et al. (2015). Our work complements the study of Stikov et al. (2015) by investigating the variation of the MR g-ratio across a population *in vivo*. The investigation of population variance was facilitated by the more rapid scanning protocol used in the present study.

While Stikov et al.'s approach was based on a time-consuming multi-shell diffusion MRI dataset to estimate the FVF for the whole brain from a NODDI protocol, we used the TFD metric that can be calculated from a standard single-shell diffusion MRI dataset (Reisert et al., 2013). However, it should be considered that the TFD-based FVF estimates might be confounded by the effect of extracellular water contamination, although the TFD methodology (Reisert et al., 2013) to some extent accounts for this effect. As a result, further investigations are required to corroborate the observed division of tracts into two groups with differing MR g–ratios. With such a confirmation the g-ratio promises to answer functionally relevant questions, e.g., to predict whether the two observed groups of tracts with different g-ratios have different conduction velocities. Additional *ex-vivo* studies similar to West et al. (2015); Stikov et al. (2015) could help to test whether TFD is a good MRI marker for FVF. Another limitation of the TFD metric is the fact that it uses a global normalization factor, which is calculated for each subject individually. As a consequence, global group differs as present in neurodegenerative diseases (Duning et al., 2010) or healthy aging (Callaghan et al., 2014) might be removed by the TFD metric. However, this limitation will not affect our current study because the age-range of the healthy group of subjects under investigation was narrow (2.8 years standard division).

To estimate MVF, Stikov et al. (2011, 2015) used a quantitative MT technique, which requires z-spectrum sampling and therefore multiple acquisitions. This makes the approach time-consuming and limits the spatial resolution of the MT maps that can be achieved within a feasible scan time (e.g., 2 mm isotropic in Stikov et al., 2011; Campbell et al., 2014). The MT saturation maps that were used in this paper can be related (Helms et al., 2008) to the solution of the binary spin-bath model of the MT FLASH sequence as derived by Pike (Pike, 1996). This indicates a correlation between the MT saturation and the transfer term, and thus the macro-molecular fraction. Although, this solution may not be sufficient for quantification of the BSB parameter (e.g., due to the qualitative difference in the TR dependence), the MT saturation measured in this study relies on a single MT-weighted FLASH image as opposed to the ~10 samples of the z spectrum used in quantitative MT, making it much faster. Together with the high SNR efficiency of the FLASH acquisition this supports a very time-efficient high isotropic resolution of 800 μm (~37 min). In addition, the MT saturation measure is largely insensitive to B1+ inhomogeneities (Helms et al., 2008; Helms, 2015). Whereas a striking artifact, attributed by the authors to B1+ inhomogeneity, is visible in Stikov et al.'s recent g-ratio paper (Stikov et al., 2015, Figure 3), we did not observe any bias in the g-ratio maps of our population due to B1+ inhomogeneities. Moreover, the MPM method is straightforward to implement and provides additional complementary information, e.g., relaxometry measures. The approach, and its sensitivity to myelination, has been demonstrated in neuroscience (Dick et al., 2012; Sereno et al., 2013; Callaghan et al., 2014) and clinical research (Freund et al., 2013; Grabher et al., 2015).

The MT and TDF measures used in this study required a calibration factor to capture MVF and FVF, respectively. The estimation of the g-ratio depends on the ratio of these calibration constants only, which we introduced as a single rescaling constant α (Equation 6) and calibrated based on g-ratio literature values in the corpus callosum of a single subject. The use of a single constant precludes the assessment of MVF and FVF separately, and reduces the model degrees of freedom.

It is beyond the scope of this paper to perform a detailed comparison between our proposed method for MVF and FVF estimation and other methods. Here, we only highlight the most salient differences, leaving more detailed comparisons in terms of precision, accuracy, and feasibility for future studies.

### Misalignment between MT and dMRI data

Another important issue in estimating the MR g-ratio from dMRI and MT maps is that the artifacts in each method need to be minimized. The most prominent artifact is caused by the susceptibility-related distortion in the dMRI dataset, which leads to a spatial mismatch between the dMRI and MT maps. The susceptibility distortions scale with the magnetic field strength, e.g., they are stronger at 3T than at 1.5T. The first MR g-ratio mapping experiment (Stikov et al., 2011) was performed at 1.5T and it could be argued that the susceptibility distortions were negligible. However, the more recent MR g-ratio mapping experiments have been done at 3T (Campbell et al., 2014; Stikov et al., 2014b), increasing the need for appropriate susceptibility-distortions correction methods. Here, we showed that if this artifact is not corrected appropriately, it strongly biases the estimated MR g-ratio map (Figure 2).

### Outlook

During the last decade quantitative MRI (qMRI) has facilitated the assessment of microstructural changes *in-vivo* (Duning et al., 2010; Fields, 2010; Meinzer et al., 2010; Zatorre et al., 2012; Freund et al., 2013; Helbling et al., 2015). Although, conventional qMRI techniques such as diffusion tensor imaging (DTI) are sensitive to microstructural changes (Kovac et al., 2009; Warnecke et al., 2010; Duning et al., 2011), they are difficult to relate to specific tissue compartments (Keller et al., 2011; Jones et al., 2013). An example is the sensitivity of DTI to microstructural changes in multiple sclerosis but its inability to distinguish demyelination from axonal degeneration (Barkhof et al., 2009). This lack of biological specificity limits the use of many conventional qMRI measures as MRI-based biomarkers. One approach to develop more specific biomarkers relies on advanced biophysical models that relate the MRI signal to the underlying microstructural characteristics, i.e., the biological characteristic of interest such as the g-ratio (Weiskopf et al., 2015).

It is well established that in addition to the axonal diameter, the g-ratio bears a direct relation to conduction velocity (Rushton, 1951; Johansen-Berg and Behrens, 2009). For example, following the theoretical analysis of Hodgkin, a deviation of 0.2 from the optimal g-ratio of *g* = 0.6, i.e., *g* = 0.4 or *g* = 0.8, would lead to an decrease in conduction time of about 30% (Hodgkin, 1964). Although, the MR g-ratio is a simplified approximation of the microscopic g-ratio (e.g., it cannot model multi-modal distribution of g-ratio within a voxel), it can provide additional relevant information in neuroscience studies. For example, the effective functional connectivity between brain areas may be more accurately determined by constructing structural connectivity maps that are weighted by the local g-ratio. These weighted connectivity maps could be particularly sensitive to those connections that are more efficient for information transfer and thus of higher functional relevance. These g-ratio weighted connectivity maps may inform brain network analyses or improve connectivity priors in functional analyses (Bullmore and Sporns, 2009; Stephan et al., 2009). In clinical research, the g-ratio maps may provide particularly sensitive and specific measures of neurodegenerative processes. This notion has been supported by the preliminary results of Stikov et al. (2014a), who showed that the MR g-ratio could distinguish newer from older multiple-sclerosis lesions, whereas the MVF and FVF maps were insensitive to the age of the lesions.

The recent insight that the MR g-ratio can also be related to an area-weighted average of g-ratios in a distribution of fibers, is another motivation for increasing the spatial resolution to reduce partial-volume effects in MR-based tractography (e.g., Roebroeck et al., 2008; Heidemann et al., 2012). Our MT maps have sub-millimeter resolution (~0.5 mm^3^ volume) and thus are well suited for minimizing partial-volume effects. However, the current spatial resolution of our g-ratio maps is restricted by the single-shell dMRI data, which were acquired at a standard resolution of ~2.3 mm isotropic. Higher spatial resolution in dMRI would help to reduce partial-volume effects in our g-ratio maps and probably reduce the high CoV toward the edge of white matter. Higher spatial resolution in dMRI can be achieved within a clinically feasible scan time using restricted-field-of-view imaging (e.g., Mohammadi et al., 2013a), multi-band (e.g., Feinberg et al., 2010), adaptive smoothing (e.g., Becker et al., 2014; Tabelow et al., 2015), and super-resolution (e.g., Ruthotto et al., 2014) techniques. Furthermore, these techniques can be combined to estimate high-resolution beyond-tensor models such as the kurtosis tensor (Jensen et al., 2005; Mohammadi et al., 2014), which can describe more complex brain microstructure (Hui et al., 2015), and thus might in the future facilitate a similar type of MR g-ratio mapping even within the cortical gray matter.

## Conclusion

We introduce a novel method for *in-vivo* g-ratio mapping using standard MRI acquisition methods. We measured whole-brain white matter g-ratio maps in a group of healthy volunteers that may serve as a reference point for future studies. We found qualitative agreement between the MR g-ratio and *ex-vivo* histological g-ratio. Although further validation studies are crucial, the MR g-ratio measure holds promise as a biomarker in neuroimaging, clinical research and diagnosis due to its improved interpretability over current MRI markers.

## Funding

The Wellcome Trust Centre for Neuroimaging is supported by core funding from the Wellcome Trust 091593/Z/10/Z. SM was supported by the Deutsche Forschungsgemeinschaft (DFG, MO 2397/1-1). The research leading to these results has received funding from the European Research Council under the European Union's Seventh Framework Programme (FP7/2007-2013)/ERC grant agreement n°616905. Open access was supported by the Wellcome Trust.

### Conflict of interest statement

The authors declare that the research was conducted in the absence of any commercial or financial relationships that could be construed as a potential conflict of interest. The Wellcome Trust Centre for Neuroimaging has an institutional research agreement with Siemens and receives support from Siemens.
